# A Practical Machine Learning Model for Predicting Neoadjuvant Response in HER2-Positive Breast Cancer

**DOI:** 10.3390/diagnostics16152374

**Published:** 2026-07-28

**Authors:** María Azmat, Lucía Graña-López, Manuel Fernández-Delgado, Eva Cernadas, Marcelino Maneiro, Cristina Núñez

**Affiliations:** 1 Centro Singular de Investigación en Tecnoloxías Intelixentes da USC (CiTIUS), Universidade de Santiago de Compostela, 15705 Santiago de Compostela, Spain; maria.azmat@usc.es (M.A.); eva.cernadas@usc.es (E.C.); 2 Radiology Department, Hospital Lucus Augusti Lugo, 27003 Lugo, Spain; lucia.grana.lopez@sergas.es; 3 Breast Pathology Group, Hospital Universitario Lucus Augusti, 27003 Lugo, Spain; 4 Institute for Research in Global Health and Sustainable Development, iTERRA, Inorganic Chemistry Department, Faculty of Sciences, Campus Terra, University of Santiago de Compostela, 27002 Lugo, Spain; marcelino.maneiro@usc.es

**Keywords:** HER2-positive breast cancer, pathological complete response, neoadjuvant therapy, breast MRI, machine learning, clinical prediction model

## Abstract

**Background/Objectives:** Pathological complete response (pCR) after neoadjuvant chemotherapy (NAC) is an important prognostic marker in HER2-positive breast cancer (BC). However, reliable pre-treatment predictors based on routinely available clinical data remain limited. This study evaluated whether clinicopathologic and baseline magnetic resonance imaging (MRI) variables could predict NAC response using classical machine learning (ML) approaches. **Methods:** A retrospective cohort of 112 patients with HER2-positive BC treated with NAC was analysed, including 57 patients who achieved pCR and 55 who did not. Fourteen pre-treatment variables were evaluated, including hormone receptor (HR) status, tumour grade, ER and PR expression, Ki67, nodal status, age, and baseline MRI characteristics. Seventy ML classifiers were compared using a fully nested leave-one-out cross-validation (LOOCV) framework. Performance was assessed using accuracy, area under the receiver operating characteristic curve (AUROC), Cohen’s kappa, sensitivity, specificity, and F1-score. **Results:** A support vector machine (SVM) with a radial basis function (RBF) kernel achieved the highest observed accuracy (82.1%) among the 70 evaluated classifiers. The corresponding AUROC was 83.3%, Cohen’s kappa was 64.2%, and sensitivity, specificity, and F1-score were 87.7%, 76.4%, and 83.3%, respectively. HR-related variables, particularly PR and ER expression, together with Ki67 and baseline MRI features, ranked among the most influential predictors. Despite relying exclusively on routinely available pre-treatment variables, the model demonstrated meaningful predictive performance. **Conclusions:** ML applied to routinely available clinicopathologic and baseline MRI variables showed promising ability to predict pCR before treatment initiation in HER2-positive BC. The proposed approach may support pre-treatment clinical risk assessment using information already generated during routine clinical assessment. Nevertheless, prospective multicentre external validation, calibration assessment, and evaluation of clinical utility are required before implementation in routine clinical practice.

## 1. Introduction

Human epidermal growth factor receptor 2 (HER2)-positive BC accounts for approximately 15–20% of BCs and is characterised by aggressive biological behaviour. The introduction of NAC combined with HER2-targeted therapy has markedly improved clinical outcomes, and pCR is now widely accepted as an early surrogate marker associated with favourable long-term prognosis [1]. Patients achieving pCR generally experience significantly better event-free and overall survival, particularly in biologically aggressive BC subtypes [2]. Nevertheless, despite major therapeutic advances, a substantial proportion of patients do not achieve pCR and remain at increased risk of disease recurrence. Moreover, patients with residual invasive disease after NAC may benefit from post-neoadjuvant treatment escalation, highlighting the clinical value of identifying likely responders and non-responders before treatment initiation [3,4,5].

Predicting response to neoadjuvant therapy remains challenging because HER2-positive BC is biologically heterogeneous. Several routinely available clinicopathologic variables, including HR status, estrogen receptor (ER) and progesterone receptor (PR) expression, proliferative activity (Ki-67), tumour grade, and nodal status, have been associated with treatment response. However, the predictive value of individual biomarkers remains limited, suggesting that more comprehensive models integrating complementary sources of information may improve patient stratification [6,7].

Breast MRI provides additional information on tumour characteristics before treatment and has become an important component of response assessment. Previous studies have demonstrated that baseline MRI features, particularly diffusion-weighted imaging (DWI) and ADC measurements, may contribute to predicting pathological response. Furthermore, multiparametric MRI approaches combining morphologic and functional information have shown improved predictive performance compared with isolated imaging biomarkers [8,9,10,11].

In recent years, ML has emerged as a promising strategy for integrating clinicopathologic and imaging information into predictive models. Several studies have reported encouraging results by combining routine clinical variables with MRI-derived features or radiomic descriptors to predict pCR following neoadjuvant therapy in HER2-positive BC [12,13,14]. However, many published models rely on complex radiomic pipelines or advanced image-processing techniques that limit their applicability in everyday clinical practice, highlighting the need for simpler and more reproducible prediction models [11].

Therefore, the aim of the present study was to develop and evaluate an ML model for predicting pCR after NAC in HER2-positive BC using exclusively routinely available pre-treatment clinicopathologic and baseline MRI variables. By restricting the model to information obtained during the standard diagnostic work-up, we sought to develop a practical and reproducible prediction tool that could support future individualised treatment planning and risk stratification.

## 2. Materials and Methods

### 2.1. Study Population

Patients were retrospectively recruited between March 2013 and January 2025 at Hospital Universitario Lucus Augusti (HULA), Lugo, Spain. All patients received anthracycline- and/or taxane-based NAC combined with HER2-targeted therapy according to institutional protocols in force at the time of treatment. Of the 112 patients included, 96 (85.7%) received dual HER2 blockade with trastuzumab plus pertuzumab, generally in combination with anthracycline- and/or taxane-based chemotherapy, whereas 16 (14.3%) received trastuzumab-based therapy without pertuzumab. The variability in treatment regimens reflects the evolution of clinical practice during the study period and individualised treatment decisions.

### 2.2. Materials

A total of 14 pre-treatment predictor variables were selected before model development (Table 1). Predictor selection was based on two complementary criteria: (i) previous evidence supporting the variables’ association with pCR after neoadjuvant therapy in HER2-positive BC and (ii) their routine availability during the standard pre-treatment diagnostic work-up. Accordingly, only variables available before treatment initiation were included, while longitudinal imaging biomarkers such as ΔADC were intentionally excluded to ensure the model could be applied at the time of initial therapeutic decision-making. No data-driven feature selection procedure was performed before model training in order to preserve clinical interpretability and avoid selection bias.

Prior to model development, the dataset was reviewed for completeness. All predictor variables were complete for all included patients; therefore, no missing-data imputation or other missing-data handling procedures were required.

MRI-derived variables, including ADC measurements, lesion type, and enhancement curve, were retrospectively extracted from the original baseline MRI reports generated during routine clinical assessment by dedicated breast radiologists following the institutional breast MRI protocol and the American College of Radiology Breast Imaging Reporting and Data System (BI-RADS^®^) MRI lexicon [15]. Lesion type and enhancement curve were defined according to the BI-RADS MRI descriptors and extracted directly from the original clinical reports without additional image reinterpretation for the purposes of this study. ADC measurements were performed on DWI using the corresponding ADC maps. A manually drawn region of interest (ROI) was placed on the solid component of the primary tumour while carefully avoiding cystic, necrotic, haemorrhagic, or biopsy-related changes. All MRI examinations were acquired according to the same institutional breast MRI acquisition protocol throughout the study period to ensure consistency in image acquisition and interpretation. No additional tumour segmentation, image reinterpretation, or dedicated radiomic analysis was performed specifically for this study. Because all MRI-derived variables were retrospectively obtained from routine clinical reports, formal interobserver agreement was not evaluated. This approach was intentionally adopted to evaluate the predictive value of imaging variables routinely available in standard clinical practice, thereby maximising the transparency, reproducibility, and potential clinical applicability of the proposed ML model, in accordance with current recommendations for reporting artificial intelligence (AI) studies in medical imaging [16].

All MRI examinations were acquired according to the same institutional breast MRI acquisition protocol throughout the study period to ensure consistency in image acquisition and interpretation. All MRI measurements were performed by an experienced breast radiologist as part of routine clinical reporting. Because ADC values were retrospectively extracted from routine clinical reports, formal interobserver agreement was not evaluated.

MRI1 refers to baseline MRI performed before initiation of NAC. Only baseline MRI-derived variables were included to ensure a pre-treatment predictive framework. Note that pCR was defined as the absence of residual invasive carcinoma in both the breast and axillary lymph nodes at surgery (ypT0/is ypN0). Patients with any residual invasive disease were classified as non-pCR. The dataset includes 112 cases, each corresponding to a patient, distributed in 55 patients with partial or no-response (non-pCR) and 57 responding patients (pCR), so both classes were fairly balanced.

Because the present study exclusively included HER2-positive breast cancers, tumour subtype (luminal B HER2-positive versus HER2-enriched) was directly determined by hormone receptor (HR) status. Consequently, Feature 14 represents an alternative clinical categorisation derived from HR status rather than an independent biological variable. It was retained because this classification is routinely used in breast oncology to distinguish the two principal HER2-positive clinical subtypes. Therefore, HR status and tumour subtype should not be interpreted as independent predictors, but rather as two alternative clinical representations of the same underlying biological information.

### 2.3. Machine Learning (ML)

To explore the predictive signal across different modelling strategies, we evaluated a broad panel of 70 classical ML classifiers from several algorithmic families. This comparison was intended as a benchmarking analysis rather than as evidence of clinical superiority of a specific software implementation. Detailed classifier-level results, including the complete list of the 70 evaluated classifiers and their corresponding accuracy values, are provided in Supplementary Table S1 [17].

The dataset was standardised to zero mean and unit variance for each feature. Standardisation parameters were estimated exclusively from the training partition within each inner cross-validation loop and subsequently applied to the corresponding validation or test sample. This procedure was repeated independently for every fold to prevent information leakage. Consequently, model performance estimates were obtained using a fully nested validation framework designed to minimise information leakage.

Given the relatively small sample size (*N* = 112), classifier performance was evaluated using nested leave-one-out cross-validation (LOOCV). For each outer-loop iteration, one patient was held out as the test case, while the remaining *N* − 1 patients constituted the training set. Classifiers were trained on the training subset and evaluated on the held-out patient. This process was repeated *N* times so that each patient served once as an independent test case.

For classifiers requiring hyperparameter optimisation, an inner cross-validation loop was performed exclusively within the training data of each outer-loop iteration. The optimal hyperparameter values were selected from a list of values, reported in the Description column of Table S1, through grid search using internal training and validation subsets. The classifier with the best-performing hyperparameter configuration was subsequently retrained on the complete outer-loop training set and evaluated on the held-out test patient.

Performance metrics were calculated by comparing the true and predicted class labels across all outer-loop test predictions. Additional experiments using dummy-variable encoding for categorical features were also performed. Because these approaches consistently produced lower classification performance, they are not reported further.

The 70 classifiers were trained and evaluated independently using the same fully nested LOOCV framework, thereby avoiding information leakage and ensuring an unbiased performance estimate for each individual classifier. However, selecting the classifier with the highest observed accuracy among the 70 evaluated models may introduce a degree of selection bias because the maximum observed performance is influenced by random sampling variability. Therefore, the highest accuracy reported in this study should be interpreted as the best observed result within the evaluated classifier collection rather than as the expected performance of the selected classifier on future independent datasets.

### 2.4. Use of Artificial Intelligence

During the preparation of this manuscript, OpenAI ChatGPT (GPT-5.5, ChatGPT, OpenAI, San Francisco, CA, USA) was used exclusively to assist with language editing, improvement of grammar, readability, and scientific writing style in selected sections of the manuscript. The AI tool was not used for study design, data collection, data analysis, interpretation of results, generation of scientific conclusions, or reference selection. All outputs were critically reviewed, verified, and edited by the authors, who take full responsibility for the final content of the manuscript.

## 3. Results

### 3.1. Baseline Characteristics of the Study Cohort

The baseline clinicopathologic and MRI characteristics of the study cohort are summarised in Table 2. Categorical variables are presented as frequencies and percentages, whereas continuous variables are reported as minimum, maximum, mean, and standard deviation. Several variables showed an uneven distribution, particularly histological subtype and baseline MRI lesion type, reflecting the clinical characteristics of HER2-positive BC in routine practice. Among continuous variables, ADC displayed an approximately Gaussian distribution, whereas ER and PR expression exhibited marked skewness. The complete distributions of all evaluated variables, and of the class labels, are presented in Supplementary Figure S1.

**Figure 1:**
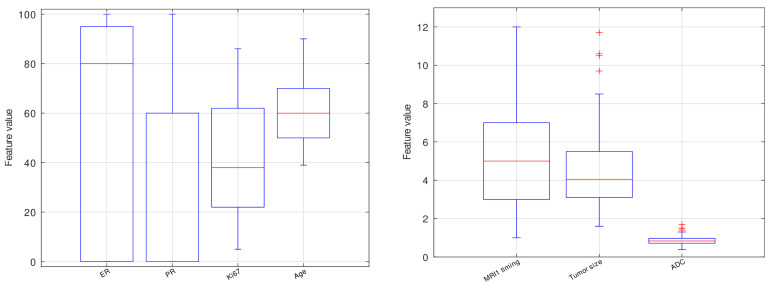
Box plots of numeric features.

The distributions of the principal continuous variables are illustrated in Figure 1. ER and PR expression showed a markedly skewed distribution, whereas ADC displayed a more symmetric distribution.

Exploratory comparison of predictor distributions according to treatment response, plotted in Supplementary Figure S2, revealed biologically plausible differences between responders (pCR in Figure S2) and non-responders (non-pCR). Among all evaluated variables, ER and PR expression showed the greatest separation between response groups, with higher expression levels observed in non-responders. In contrast, most clinicopathologic and MRI-derived variables displayed substantial overlap between classes. These descriptive analyses were performed for visualisation purposes only and were not used for feature selection or model development.

### 3.2. Comparison of ML Classifiers

The usual performance metrics for binary classification problems were evaluated, including accuracy, Cohen’s kappa, AUROC, precision, recall (sensitivity), specificity, and F1-score (Dice coefficient). Supplementary Table S1 reports the accuracy values achieved by the 70 evaluated ML classifiers used to predict response. These values range from 52.7% (NBC in Octave 9.2.0 and NB in Python 3.11) to 82.1% using SVM in Matlab R2024a.

Table 3 summarises the highest accuracy achieved by each classifier family across the evaluated programming environments, while a comprehensive comparison of all 70 classifiers is provided in Supplementary Table S1. The highest observed accuracy among the 70 evaluated classifiers was obtained by the Matlab implementation of the SVM (82.1%; 95% Wilson confidence interval: 73.8–88.3%) [18]. This value represents the best observed result within the evaluated classifier collection and should not be interpreted as an unbiased estimate of the expected predictive performance of the selected classifier. Among the remaining approaches, the extreme learning machine (ELM) achieved the highest accuracy in Octave (73.2%), the Random Feature Gaussian Kernel Classifier (RFGKC) was the second-best Matlab model (77.7%), stochastic gradient descent (SGD) achieved the best result in Python (74.1%), and random forest together with the ensemble of multilayer perceptrons (avNNet) obtained the highest accuracy in R 4.4 (70.5%). Although several of the highest-performing models were implemented in Matlab, these differences should not be interpreted as evidence that a particular programming language is intrinsically superior. Rather, the observed performance variations are more likely attributable to differences in algorithmic implementation, hyperparameter optimisation, and the characteristics of the present dataset.

This interpretation is supported by three complementary observations. First, implementations of the same SVM algorithm in different software environments produced accuracies ranging from approximately 70% to 74%, indicating some variability across implementations. Second, the difference between the best-performing SVM and the second-best classifier (RFGKC) was not statistically significant, as discussed in Section 3.4. Third, the 95% Wilson confidence interval around the observed accuracy indicates substantial uncertainty in the performance estimate. Collectively, these findings suggest that the highest observed accuracy should be interpreted with appropriate caution and regarded as the best observed result among the evaluated classifiers rather than as the expected performance of the selected model on future independent datasets.

### 3.3. Performance of the Highest-Performing Classifier Among the Evaluated Models

Table 4 reports the confusion matrix (T = true, F = false, N = negative or non-pCR, and P = positive or pCR) corresponding to the classifier that achieved the highest observed accuracy among the 70 evaluated classifiers, namely the Matlab implementation of the SVM with an RBF kernel, using nested LOOCV.

This classifier achieved the highest observed accuracy (82.1%) among the 70 evaluated classifiers, together with a Cohen’s kappa of 64.2%, which, according to [19], indicates good agreement between true and predicted responses. As discussed in the previous section, this result should be interpreted as the best observed performance among the evaluated classifiers rather than as an unbiased estimate of the expected predictive performance of the selected classifier. Precision (=100·TP/(TP + FP)) and recall (=100·TP/(TP + FN)) were 79.4% and 87.7%, respectively, whereas specificity (=100·TN/(TN + FP)) was 76.4%. The corresponding F1-score (Dice coefficient) was 83.3%. The classifier produced more false-positive predictions (13) than false-negative predictions (7), corresponding to a false-positive rate (FPR) of 23.6% and a false-negative rate (FNR) of 7.6%.

**Figure 2:**
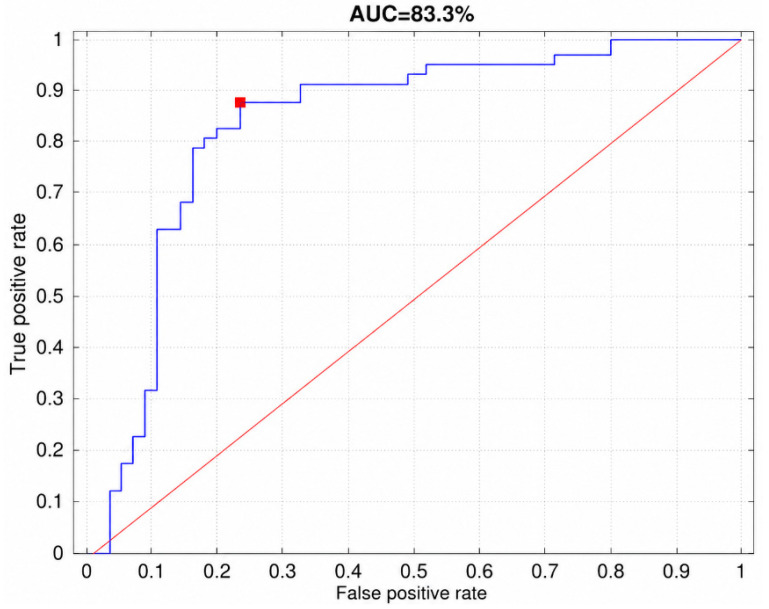
ROC (blue line) and working point (red square) of the best classifier.

Figure 2 shows the receiver operating characteristic (ROC) curve achieved by the Matlab implementation of the SVM. The ROC curve is clearly separated from the diagonal line representing random classification, and the operating point is located close to the upper-left corner, indicating a favourable balance between sensitivity and specificity. Consistent with this visual assessment, the classifier achieved a high AUROC of 83.3%.

### 3.4. Statistical Comparison of Classifier Performance

Table 5 reports the *p*-values of three statistical tests comparing the classifier achieving the highest observed accuracy (SVM, 82.1%) with the classifier achieving the second-highest observed accuracy (RFGKC, 77.7%) and with a baseline classifier (Logreg, 70.5%). These tests are the two-proportions test, the resampled paired *t*-test and McNemar’s test. The null hypothesis is that the difference between SVM and RFGKC or Logreg has a mean equal to zero. For a tolerance of 5%, this hypothesis is accepted (i.e., the difference is not statistically significant) if *p* > 0.05 and rejected (i.e., the difference is statistically significant) otherwise. When comparing the classifier achieving the highest observed accuracy (SVM) with RFGKC, *p* > 0.05 for all three tests; therefore, the null hypothesis was not rejected, because the observed accuracy difference (82.1% − 77.7% = 4.4 percentage points) was not statistically significant given the limited sample size (*N* = 112). However, all three *p*-values were below 0.05 for Logreg, indicating that the difference relative to SVM (82.1% − 70.5% = 11.6 percentage points) was statistically significant in this dataset.

### 3.5. Descriptive Calibration Assessment and Feature Importance

**Figure 3:**
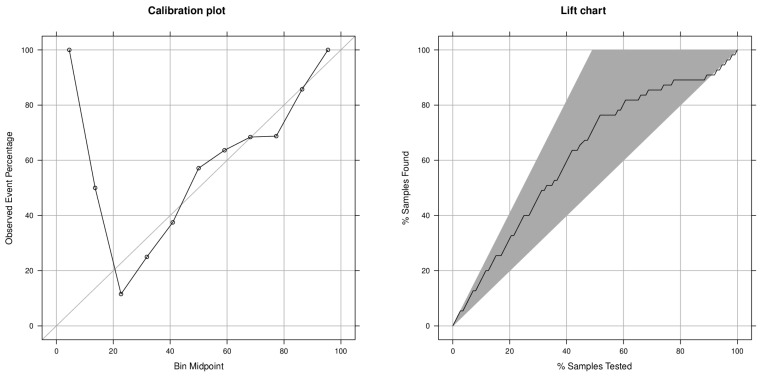
Calibration plot and lift chart of SVM.

Figure 3 shows the calibration plot (left) and lift chart (right) of the SVM. The calibration plot reports the predicted probabilities versus true probabilities. In a well-calibrated model, the points should lie close to the diagonal. This happens for all the points except for the two on the left, indicating overall good agreement despite some deviations. The lift chart plots the percentage of cases predicted as positive versus the per-centage of true-positive cases. An ideal lift chart is characterised by a curve located close to the left boundary of the shaded triangle. In this case, most of the line is near the left side of the triangle, but the curve progressively shifts towards the right for horizontal coordinates above 80, corresponding to the points far from the diagonal in the calibration plot.

**Figure 4:**
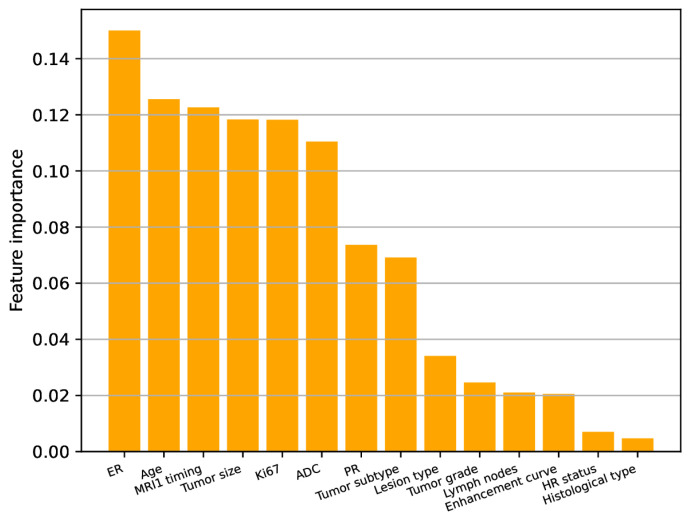
Importance of features according to the exploratory random forest analysis. Because tumour subtype is derived from HR status in this HER2-positive cohort, the contributions of these two variables should not be interpreted as independent.

To facilitate the clinical interpretation of the predictors included in the final model, an exploratory feature importance analysis was performed using a random forest classifier trained on the same set of clinicopathologic and MRI variables. Because the final prediction model was based on an SVM with an RBF kernel, this analysis was not intended to explain the internal decision process of the SVM but rather to provide an approximate ranking of predictor relevance. Furthermore, impurity-based feature importance measures are known to favour continuous or high-cardinality variables and should therefore be interpreted as descriptive and exploratory. The resulting feature importance profile is presented in Figure 4.

According to the random forest feature importance ranking, ER, age, MRI1 timing, tumour size, Ki67, ADC and PR were among the most influential predictors. The prominent contribution of ER, Ki67 and PR is consistent with the class-stratified distributions shown in Supplementary Figure S2.

Because tumour subtype is directly derived from HR status in a cohort exclusively composed of HER2-positive breast cancers, Feature 14 should not be interpreted as an independent predictor. Its inclusion simply reflects the alternative clinical categorisation routinely used in breast oncology and explains its overlapping contribution with HR-related variables in the exploratory feature importance analysis.

## 4. Discussion

The present study demonstrates that routinely available pre-treatment clinicopathologic and breast MRI variables contain clinically relevant predictive information to identify patients with HER2-positive breast cancer who are unlikely to achieve pathological complete response (pCR) after neoadjuvant systemic therapy. Rather than proposing a tool for immediate treatment adaptation, our findings indicate that machine learning may support pre-treatment clinical risk assessment using data already generated during standard clinical assessment. This practical approach is aligned with the growing interest in developing clinically interpretable and implementation-oriented machine learning models that can be integrated into routine oncological workflows without requiring specialised image-processing pipelines or extensive molecular profiling [6,12,14,19,20,21]. Among the 70 evaluated classifiers, the highest observed accuracy was 82.1%. However, this value represents the best observed performance within the evaluated classifier collection and should therefore be interpreted with appropriate caution. The observed variability across software implementations, the absence of statistically significant superiority over the second-best classifier, and the corresponding confidence interval indicate that this result should not be interpreted as an unbiased estimate of the expected predictive performance of the selected classifier.

The exploratory feature importance analysis presented in the Results section provides a biologically plausible ranking of predictor relevance and is broadly consistent with established predictors of response in HER2-positive disease. Because the feature importance analysis was derived from a random forest model rather than the final SVM classifier, the results should be interpreted as descriptive instead of as a direct explanation of the SVM decision process. Furthermore, impurity-based importance measures are known to favour continuous predictors and should therefore be interpreted cautiously. HR expression, particularly ER and PR, emerged among the most influential variables, consistent with previous evidence indicating that HR-negative or HR-low HER2-positive tumours are more likely to achieve pCR following anti-HER2 neoadjuvant therapy. It should also be noted that the tumour subtype variable included in the model corresponds to the routine clinical classification of HER2-positive tumours into luminal B HER2-positive and HER2-enriched disease, which is directly derived from HR status in this cohort and therefore should not be interpreted as an independent source of predictive information. MRI1 timing also contributed to prediction, although its importance is more likely to reflect differences in imaging workflow, clinical scheduling, and temporal changes in treatment practice than intrinsic tumour biology [8].

Our findings should be interpreted within the context of the evolving management of HER2-positive BC. Patients included in this study were recruited between March 2013 and January 2025, a period during which neoadjuvant treatment progressively evolved following the incorporation of dual HER2 blockade into routine clinical practice. Consequently, most patients in the present cohort received trastuzumab plus pertuzumab (96/112, 85.7%), whereas a smaller proportion received trastuzumab without pertuzumab (16/112, 14.3%), reflecting the standard of care at the time of treatment as well as individual clinical considerations. Therefore, the therapeutic composition of the present cohort should be considered when interpreting the performance and generalisability of the proposed prediction model. Large clinical trials have established both the prognostic relevance of pCR and the therapeutic implications of residual disease, demonstrating that patients who do not achieve pCR have a poorer prognosis but may benefit from post-neoadjuvant treatment escalation [2,5]. Consequently, accurate pre-treatment identification of patients unlikely to achieve pCR remains clinically valuable, particularly in view of the therapeutic opportunities currently available for patients with residual disease. In this context, predictive ML models have the potential to support early risk stratification before treatment initiation, provided that their clinical usefulness is assessed not only by discrimination metrics but also by the consequences of potential misclassification. Prediction models should therefore be judged not only by their discrimination performance but also by the potential clinical consequences of incorrect treatment stratification.

Beyond the available clinical evidence, our findings are also consistent with previous ML studies demonstrating that the integration of clinicopathologic and MRI-derived information improves prediction of pCR in HER2-positive BC [10,12,14]. However, many published models rely on high-dimensional radiomic features or complex image-processing pipelines that may limit reproducibility and hinder implementation in routine clinical practice. These challenges are common to many AI applications in breast imaging, where external validation, transparency, regulatory approval and integration into radiological workflows remain major barriers to widespread clinical implementation [22]. In contrast, the present model deliberately prioritised routinely available pre-treatment variables to maximise interpretability, reproducibility and potential clinical applicability, while maintaining clinically meaningful predictive performance [10,12]. This pragmatic strategy is increasingly recognised as essential for successful clinical translation, as recent reviews have highlighted that successful implementation of AI in breast imaging requires robust validation, transparency, reproducibility, and integration into routine clinical workflows [23,24].

Another important consideration for future clinical implementation is model interpretability. Although highly complex AI algorithms may achieve excellent predictive performance, their adoption in clinical practice depends increasingly on the ability to provide transparent and understandable predictions that clinicians can confidently incorporate into therapeutic decision-making. Consequently, prediction models based on routinely available clinicopathologic and imaging variables may facilitate clinician acceptance while preserving clinically meaningful predictive accuracy [25].

From a translational perspective, the principal advantage of the proposed model is its exclusive reliance on routinely available clinicopathologic and MRI variables, making implementation considerably simpler than approaches requiring radiomics pipelines or molecular assays. Given its current error profile, however, the model should primarily be viewed as a tool for escalation-oriented risk stratification rather than treatment de-escalation. Patients predicted to have a high probability of residual disease could potentially benefit from closer imaging surveillance, earlier multidisciplinary review or prioritisation for clinical trials. In contrast, any application supporting treatment reduction would require prospective external validation, calibration analysis and optimisation of decision thresholds to minimise false responder classifications before safe clinical implementation [18,26]. This emphasis on practicality and clinical interpretability represents one of the principal distinguishing features of the proposed model.

Beyond external validation, future studies should also assess the robustness of the proposed model under real-world conditions, including variability in MRI acquisition protocols, patient demographics, and institutional clinical practice. Improving the transportability and generalisability of prediction models across different healthcare systems has become one of the major priorities for the successful translation of AI into routine BC care. These aspects are particularly relevant for models intended for widespread clinical implementation, where consistent performance across diverse imaging environments is essential [27,28].

The relatively modest contribution of baseline MRI variables (MRI1 timing, lesion type, tumour size, ADC and enhancement curve) in the present model may reflect the deliberate use of simple, routinely acquired imaging descriptors rather than advanced quantitative imaging biomarkers. Previous studies have shown that longitudinal MRI information, particularly early treatment-induced changes in DWI and ADC, together with standardised radiomic features, can improve prediction of pCR beyond baseline imaging alone [10,12,18,29]. Nevertheless, incorporation of these advanced imaging biomarkers should be carefully balanced against the need for reproducibility, harmonisation of image acquisition and processing, and external transportability across institutions. Future versions of the model could therefore benefit from integrating longitudinal imaging information while preserving the simplicity and clinical applicability that represent the principal strengths of the present approach [8,10,12,30].

This study presents several important strengths. The predictive model was developed exclusively from routinely available pre-treatment clinicopathologic and MRI variables, increasing its potential applicability in routine clinical practice while avoiding the need for dedicated radiomics pipelines or molecular profiling. This deliberate design improves reproducibility and facilitates future implementation in centres with different levels of technological resources. In addition, the use of a fully nested validation framework reduced the risk of optimistic bias associated with hyperparameter optimisation and provided an unbiased performance estimate for each individual classifier. However, because the final model was selected as the highest-performing classifier among the 70 evaluated algorithms, some optimism due to this model-selection process cannot be completely excluded. Another strength is that the cohort spans a relatively long recruitment period (March 2013–January 2025), reflecting the real-world evolution of neoadjuvant HER2-targeted therapy in routine clinical practice. Consequently, by the end of the recruitment period, most patients (96/112, 85.7%) received the current standard dual HER2 blockade with trastuzumab plus pertuzumab, whereas 16 patients (14.3%) received trastuzumab-based therapy without pertuzumab, reflecting changes in treatment availability and individualised clinical decision-making over time.

Nevertheless, several limitations should be acknowledged. The study was conducted in a relatively small single-centre cohort. Calibration was explored descriptively using an internal calibration plot but was not formally quantified using calibration metrics (e.g., calibration slope or intercept) or evaluated in an external validation cohort. Consequently, the predicted probabilities should be interpreted with caution, and the proposed model should currently be regarded as a clinical decision-support tool rather than a standalone risk prediction model. Furthermore, because both quantitative (ADC) and qualitative MRI variables were retrospectively extracted from routine clinical practice, formal interobserver reproducibility was not assessed. The inclusion of patients treated with different HER2-targeted regimens may also have influenced response rates and should therefore be considered when extrapolating the model to populations treated exclusively with contemporary therapeutic protocols. Finally, future multicentre studies following current reporting recommendations, including Transparent Reporting of a Multivariable Prediction Model for Individual Prognosis or Diagnosis + Artificial Intelligence (TRIPOD+AI) and Checklist for Artificial Intelligence in Medical Imaging (CLAIM), together with formal external validation, calibration assessment and evaluation of clinical utility, will be essential before clinical implementation [16,21]. Future validation studies should also evaluate the robustness of the proposed model across different institutions, MRI acquisition protocols and patient populations, since dataset shift remains one of the principal obstacles limiting the generalisability of AI models in medical imaging [31].

## 5. Conclusions

Among the 70 evaluated classifiers, the highest observed accuracy (82.1%) was obtained using a support vector machine with a radial basis function (RBF) kernel. However, this value represents the best observed performance among the evaluated classifiers and should not be interpreted as an unbiased estimate of the expected predictive performance of the selected model. Consequently, the proposed model should currently be regarded as a promising proof-of-concept requiring external validation in independent multicentre cohorts before clinical implementation.

## Figures and Tables

**Table 1:** List of features: name, description, type and range of values.

No.	Feature	Description	Type: Values, Min/Max, Mean/Std
1	HR status	Hormone receptor expression	Discrete: 0 = HR-negative; 1 = HR-positive
2	Histological type	Carcinoma subtype	Discrete: invasive ductal carcinoma (IDC), invasive lobular carcinoma (ILC), mucinous, micropapillary, apocrine
3	Tumour grade	Histological differentiation	1 = low; 2 = intermediate; 3 = high
4	ER	Estrogen receptor	Percentage (0–100%)
5	PR	Progesterone receptor	Percentage (0–100%)
6	Ki67	Proliferation index	Percentage (5–86%)
7	Lymph nodes (US)	Suspicious lymph nodes on ultrasound (US)	Discrete: 0 = absence; 1 = presence
8	Age	Age of the patient (years)	Integer (39–90)
9	MRI1 timing	Time since baseline MRI before treatment	Integer (1–12)
10	Lesion type (MRI1)	Mass/non-mass	Discrete: 0 = non-mass; 1 = mass; 2 = both
11	Tumour size (MRI1)	Tumour size on baseline MRI (cm)	Real (1.6–11.7)
12	ADC (MRI1)	ADC on baseline MRI	Real (0.38–1.7)
13	Enhancement curve (MRI1)	DCE kinetic pattern	Discrete: 1 = progressive; 2 = plateau; 3 = washout
14	Tumour subtype	Clinical subtype within HER2-positive BC (derived from HR status)	Discrete: 1 = LB HER2-positive; 2 = HER2-enriched

**Table 2:** Percentage of patients with each value of discrete features. For each numeric feature, the minimum, maximum, mean and standard deviation are also reported.

**Discrete Features**					
**No.**	**Feature**	**Value (Population in %)**			
1	HR status	Positive (66.1%), negative (33.9%)			
2	Histological type	IDC (92%), ILC (4.5%), mucinous (1.8%), micropapillary (0.9%), apocrine (0.9%)			
3	Tumour grade	1 (12.5%), 2 (69.6%), 3 (17.9%)			
7	Lymph nodes (US)	Absence (47.3%), presence (52.7%)			
10	Lesion type (MRI1)	Non-mass (22.3%), mass (70.5%), mixed (7.1%)			
13	Enhancement curve (MRI1)	1 (progressive) (6.2%), 2 (plateau) (25%), 3 (washout) (68.8%)			
14	Subtype	HER2-enriched (33.9%), LB HER2-positive (66.1%)			
**Numeric Features**					
**No.**	**Feature**	**Minimum**	**Maximum**	**Mean**	**Std. Dev.**
4	ER (%)	0	100	52.7	43.964
5	PR (%)	0	100	26.7	37.089
6	Ki67 (%)	5	86	43.3	24.236
8	Age (years)	39	90	60.9	12.108
9	MRI1 timing	1	12	5.1	2.518
11	Tumour size (MRI1)	1.6	11.7	4.6	2.021
12	ADC (MRI1)	0.38	1.7	0.9	0.225

All MRI-derived variables correspond to MRI1. No longitudinal imaging features (e.g., changes between MRI1 and mid-treatment MRI) were included. Percentages may not sum to 100% due to rounding.

**Table 3:** Best accuracy achieved by a classifier of each language. Each line corresponds to a family, e.g., 1st line in Octave is the best Bayesian classifier; see Table S1. The best overall result is in bold.

Language	Classifier	Accuracy (%)	Language	Classifier	Accuracy (%)
**Octave**	ANBC	66.9	**Python**	Cboost	69.6
	RDA	73.2		Ctree	58.0
	ELM	73.2		KNN	70.5
	DKP	72.3		LDA	68.7
	Logreg	69.6		MLP	67.0
	Wiener-Hopf	68.7		SGD	74.1
**Matlab**	Subspace	74.1		SVM	70.5
	Ctree	68.7		NB	52.7
	DLDA	68.7		AvNNet, RF	70.5
			**R**		
	PNN	72.3		Nnet	64.3
	LASSO, Logreg	70.5		Ctree	68.7
	KNN	67.9		FDA, LDA	68.7
	**SVM**	**82.1**		KNN	69.6
	RFGKC	77.7		NB	70.0

**Table 4:** Confusion matrix achieved by the SVM implemented in Matlab.

		Predicted Response	
		Non-pCR	pCR
**True** **Response**	**Non-pCR**	(TN) **42**	(FP) 13
	**pCR**	(FN) 7	(TP) **50**

**Table 5:** The *p*-values of three statistical tests comparing the accuracies of SVM and RFGKC.

	Two-Proportions	Resampled Paired *t*-Test	McNemar’s Test
**RFGKC**	0.2021	0.2268	0.3320
**Logreg**	0.0196	0.0025	0.0059

## Data Availability

The datasets generated and/or analysed during the current study are not publicly available due to ethical and privacy restrictions associated with patient data but are available from the corresponding authors on reasonable request and subject to approval by the relevant ethics committee and institutional regulations.

## Supplementary Materials

The following supporting information can be downloaded at: https://www.mdpi.com/article/10.3390/diagnostics16152374/s1, Figure S1: Distributions of clinicopathologic and baseline MRI features; Figure S2: Class-stratified feature distributions according to pathological response (pCR versus non-pCR); Table S1: Complete list of the 70 machine learning classifiers evaluated and their classification performance.

## Abbreviations

The following abbreviations are used in this manuscript:

ADC	Apparent Diffusion Coefficient
ACRIN	American College of Radiology Imaging Network
AI	Artificial Intelligence
ANBC	Averaged n-Dependence Bayesian Classifier
AUROC	Area Under the Receiver Operating Characteristic Curve
BC	Breast Cancer
BI-RADS	Breast Imaging Reporting and Data System
CEIm-G	Comité de Ética de la Investigación con Medicamentos de Galicia
CLAIM	Checklist for Artificial Intelligence in Medical Imaging
DCE-MRI	Dynamic Contrast-Enhanced Magnetic Resonance Imaging
DLDA	Diagonal Linear Discriminant Analysis
DWI	Diffusion-Weighted Imaging
ELM	Extreme Learning Machine
ER	Estrogen Receptor
FDA	Flexible Discriminant Analysis
HER2	Human Epidermal Growth Factor Receptor 2
HR	Hormone Receptor
IDC	Invasive Ductal Carcinoma
ILC	Invasive Lobular Carcinoma
KNN	K-Nearest Neighbours
LASSO	Least Absolute Shrinkage and Selection Operator
LDA	Linear Discriminant Analysis
LOOCV	Leave-One-Out Cross-Validation
ML	Machine Learning
MRI	Magnetic Resonance Imaging
NAC	Neoadjuvant Chemotherapy
NB	Naïve Bayes
NBC	Naïve Bayes Classifier
pCR	Pathological Complete Response
PNN	Probabilistic Neural Network
PR	Progesterone Receptor
RBF	Radial Basis Function
RDA	Regularised Discriminant Analysis
RFGKC	Random Feature Gaussian Kernel Classifier
ROI	Region of Interest
SGD	Stochastic Gradient Descent
SVM	Support Vector Machine
TRIPOD+AI	Transparent Reporting of a Multivariable Prediction Model for Individual Prognosis or Diagnosis + Artificial Intelligence
US	Ultrasound
