# Supplementary material for: A Practical Machine Learning Model for Predicting Neoadjuvant Response in HER2-Positive Breast Cancer
Source: Diagnostics (Basel). 2026 Jul 28;16(15):2374. doi: 10.3390/diagnostics16152374 (PMC13465032; doi:10.3390/diagnostics16152374)
Supplement: Supplementary file 1 [file diagnostics-16-02374-s001.zip › diagnostics-4413728-supplementary.pdf]

Supplementary material

A Practical Machine Learning Model for Predicting Neoadjuvant Response in HER2-Positive Breast Cancer

Maria Azmat[1‡], Lucía Graña-López[2,3‡], Manuel Fernández-Delgado[1], Eva Cernadas[1], Marcelino Maneiro[4], Cristina Núñez[4]

[1] Centro Singular de Investigación en Tecnoloxías Intelixentes da USC (CiTIUS), Universidade de Santiago de Compostela, 15705 Santiago de Compostela, Spain

[2] Radiology Department, Hospital Lucus Augusti Lugo, Lugo 27003, Spain

[3] Breast Pathology Group, Hospital Universitario Lucus Augusti-IDIS, Lugo, 27003, Spain

[4] Inorganic Chemistry Department, Faculty of Sciences, Campus Terra, University of Santiago de Compostela, 27002 Lugo, Spain

[‡]These two authors share first authorship

This Supplementary Material provides additional methodological details and descriptive visualisations of feature distributions and class-stratified patterns that complement the analyses presented in the main manuscript.

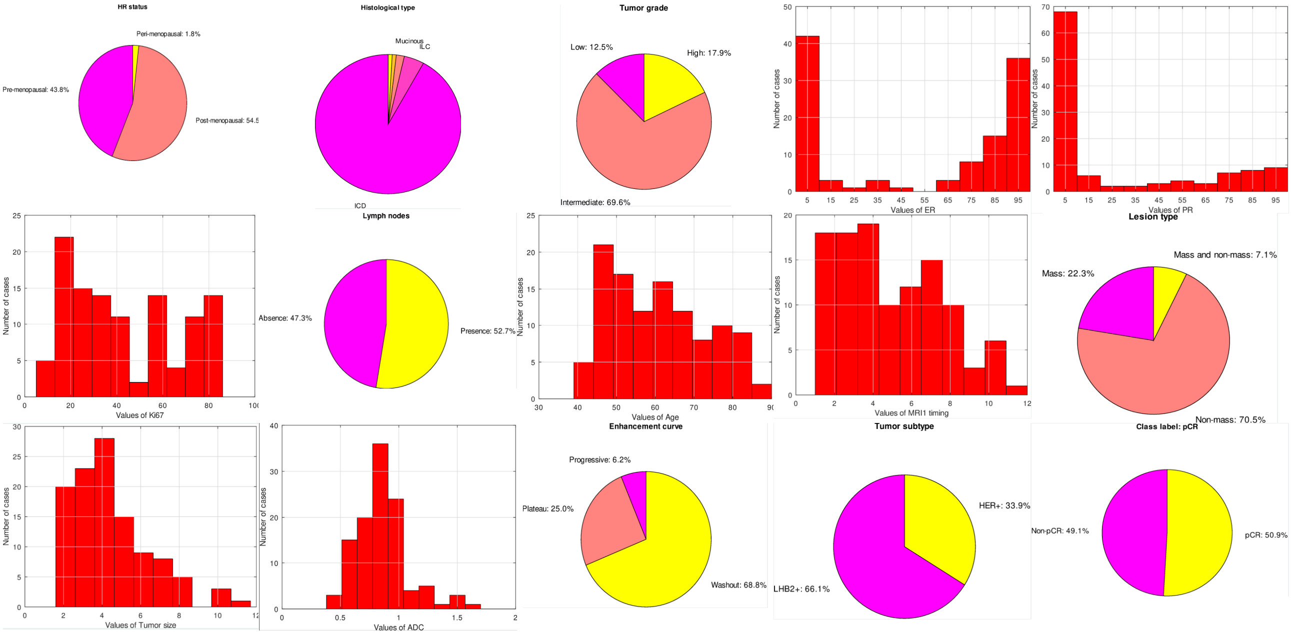

Supplementary Figure S1. Distributions of clinicopathologic and baseline MRI features included in the analysis and of class label (pCR). Discrete variables are shown as pie charts, and numeric variables

as histograms. Several categorical features are imbalanced, reflecting the real-world distribution of HER2-positive breast cancer in routine clinical practice, particularly histological type and lesion type on baseline MRI (MRI1). Among numeric variables, apparent diffusion coefficient (ADC) shows an approximately Gaussian distribution, whereas hormone receptor expression (ER and PR) displays marked skewness. Histological type shows ICD, ILC, mucinous, micropapillary and aprocrine according the percentages in Table 2 of the paper.

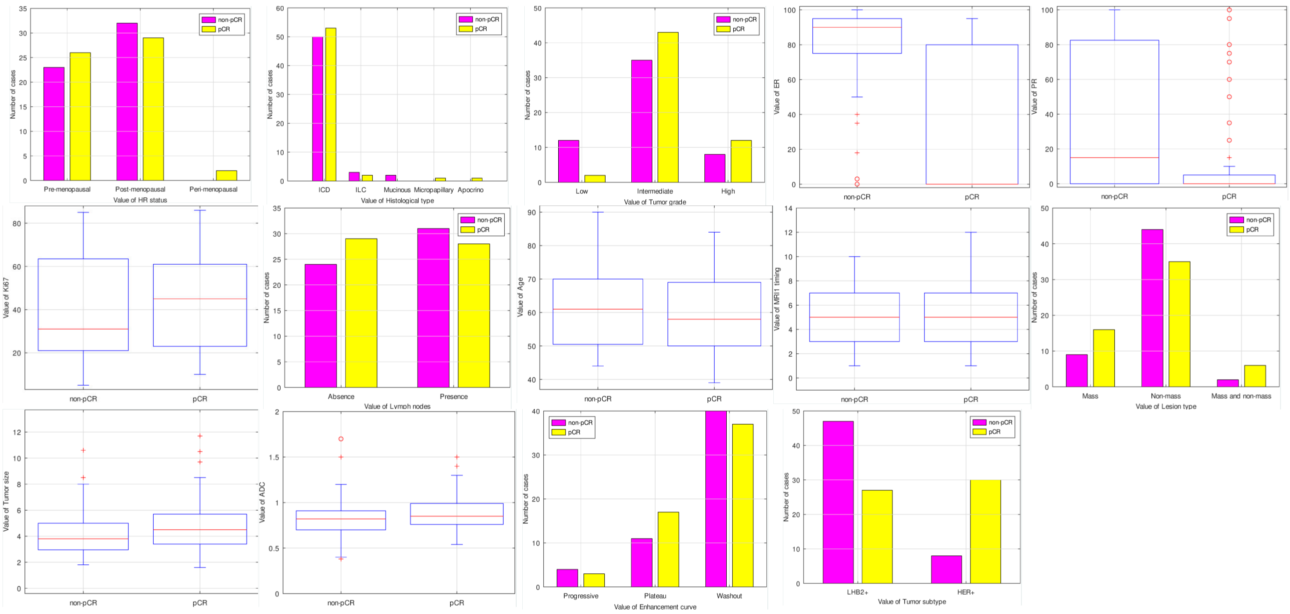

Supplementary Figure S2. Class-stratified distributions of clinicopathologic and baseline MRI features according to pathological response to neoadjuvant chemotherapy: responders (pCR) or non-responders (non-pCR). Discrete variables are displayed as bar plots showing the number of patients in each response class for each feature value, whereas numeric variables are shown as box plots stratified by response class. Estrogen (ER) and progesterone (PR) receptor expression exhibit the most pronounced separation between responders and non-responders, with higher values observed among non-responders. Other variables demonstrate more overlapping distributions between classes. These visualisations are descriptive and were not used for feature selection.

List of classifiers

Table S1 lists the 70 ML classifiers used in the experiments for the classification of response. These classifiers have been implemented in four ML computing frameworks: Octave (22 classifiers), using the NaN package through the *train_sc* function; Matlab (21), using the Statistics and Machine Learning Toolbox; Python (14), using the Scikit-Learn module; and R (13), using different packages reported in the *Description* column. The classifiers are grouped by families of related methods, that are similar in the four frameworks: Bayesian methods, discriminant analysis, neural networks, support

vector machines, linear and logistic regression, nearest neighbors, decision trees and other methods. The *Description* column also report the tunable hyperparameters and the values used in grid search, for those classifiers that require tuning. The *Accuracy* column of Table S1 reports the accuracy values achieved by each classifier. This accuracy ranges from 52.68% to 82.14%, although most of the methods (43) achieve values between 60% and 70%, and 15 methods are between 70% and 80%. Many methods of different frameworks and families achieve the same accuracy value, e.g. 15 methods achieve 68.75%, 7 methods achieve 66.96% and 6 methods achieve 67.86%.

Table S1: List of the 70 ML classifiers used in this work, grouped by language and family, with their accuracy values and values of the tunable hyperparameters, where *a*:*b*:*c* means the range of values from *a* to *b* with step *c* and *a*:*b* is the same with step 1. The values *N*, *I* and *C* denote the no. of train patterns, inputs and classes, respectively.

Language	Family	Classifier	Description	Accuracy (%)
**Octave**<br>NaN Package<br>train_sc function	Bayesian	ANBC	Averaged n-Dependence Bayesian Classifier	66.96
		NBC	Naive Bayes classifier	52.68
	Discriminant analysis	LDA	Linear discriminant analysis	69.64
		RDA	Regularized discriminant analysis: regularization=$\{2^i : i$=-20:2:5; λ=0:0.1:1	73.21
	Neural networks	PLA	Perceptron learning algorithm	57.14
		MLP1	Multilayer perceptron neural network: no. *h* of hidden neurons: $h=h_0:\Delta h:h_1$, h0=$\max(1,\lfloor h_1/H \rfloor)$, H=10, $\Delta h=\max(1,\lfloor (h_1-h_0)/H \rfloor)$; $h_1=\max(1,\lfloor N/(I+C) \rfloor)$	68.75
		MLP2	MLP network [i]: equal to MLP1	71.43
		ELM	Extreme learning machine: equal to MLP1	73.21
	Support vector machine	LSVM	Support vector machine, linear kernel: regularization λ=$\{2^i$: i=-5:2:15}	68.75
		SVM	LibSVM, support vector machine, RBF kernel [ii]: λ equal to LSVM, spread γ=$\{2^i$: i=-15:2:6}	69.64
		DKP	Direct kernel perceptron [iii]: γ equal to SVM	72.32
		PSVM	Proximal SVM [iv]: ν (NU)=$\{2^i$: i=-5:2:5}	68.75
		PPSVC	Proximal parametric-margin SV classifier [v]: γ equal to SVM, ν equal to PSVM	68.75
	Linear and logistic regression	PLS	Partial least squares linear regression	68.75
		REG	Regression analysis	68.75
		LMS	Least mean squares	57.14
		Logreg	Logistic regression: regularization λ=$\{2^i$: i=-10:1:10}	69.64
		Adaline	Adaptive linear classifier	57.14
	Other	Wiener-Hopf	Wiener-Hopf equation	68.75
		Winnow2	Winnow algorithm [vi]	57.14
		GRB	Gaussian radial basis function	54.46
		GDBC	General distance based classifier	54.46
**Matlab**<br>Statistics and Machine Learning Toolbox	Ensembles<br>(*fitcensemble* function)	Adaboost	Adaptive boosting: no. trees T=10:s:M, M=N/2, $s=\lfloor (M$-10)/$V \rceil$, V=20	65.18
		Bagging	Bootstrap aggregating: equal to Adaboost	66.96
		Gentleboost	Gentle adaptive boosting: equal to Adaboost	63.39
		Logitboost	Adaptive logistic regression: equal to Adaboost	67.86
		Lpboost	Linear programming boosting: equal to Adaboost	58.93
		RF	Random forest: no. trees T=5:5:31; no. random selected features F=3:I	68.75
		Robustboost	Robust boosting: equal to Adaboost	60.71
		Rusboost	Random undersampling boosting: no. trees T=10:s:M, M=N/2, $s=\lfloor (M$-10)/$V \rceil$, V=20; learning rate={0.01, 0.3, 0.05, 0.07, 0.1, 0.3, 0.5}	69.64
		Subspace	Random subspace: no. trees T equal to Adaboost; no. features F=1:I	74.11
		Totalboost	Totally corrective boosting: equal to Adaboost	62.50

	Decision trees	Ctree	Classification tree	68.75
	Discriminant analysis	DLDA	Diagonal linear discriminant analysis	68.75
		QDA	Quadratic LDA	67.86
	Neural networks	MLP	Multilayer perceptron neural network (*fitcnet* function): no. h of hidden neurons: $h=h_0:\Delta h:h_1$, h0=max(1,$\lfloor h_1/H \rfloor$), H=10, Δh=max(1,$\lfloor (h_1-h_0)/H \rfloor$); h_1=max(1,$\lfloor N/(I+C) \rfloor$)	64.29
		PNN	Probabilistic neural network: RBF spread $\sigma=\{2^i : i=-20:2:20\}$	72.32
	Linear and logistic regression	LASSO	Least absolute shrinkage and selection operator regression: regularization $\lambda=\{10^i : i=-7:0.5:-1\}$	70.54
		Logreg	Logistic regression	70.54
	Nearest neighbors	KNN	K-Nearest neighbor classifier: no. of neighbors K=1:2:15	67.86
	Support vector machine	SVM	Support vector machine, RBF kernel: regularization $\lambda=\{2^i : i=-5:2:15\}$, spread $\gamma=\{2^i : i=-15:2:6\}$	**82.14**
	Other	UF2DC	Ultra-fast 2D classification [vii]	66.96
		RFGKC	Random feature Gaussian kernel classification: regularization $\lambda=\{2^i : i=-5:2:15\}$, no. dimensions=$\{2^i : i=5:10\}$	77.68
**Python** Scikit-learn	Ensembles	Adaboost	Adaptive boosting: no. trees T=10:10:50, learning rate=0.1:0.5:2	67.86
		Bagging	Bootstrap aggregating: no. trees T=10:10:50, max. samples=1:11	58.04
		Cboost	Catboost (Dorogush, 2018) [viii]: no. trees T=10:5:50, learning rate={0.1, 0.2, 0.5, 0.7, 1}	69.64
		GBM	Gradient boosting machine: no. trees T=50:50:200, max. depth={1, 3, 6, 9}	66.96
		RF	Random forest: no. trees T=5:5:30, no. random selected features F=3:2:I	66.96
	Decision trees	Ctree	Classification tree: criterion={Gini, Entropy}, splitter={Best, Random}, max. features F={None, 3, 4, I, I/4, I/2, $\sqrt{I}$, $\log_2 I$ }	58.04
	Nearest Neighbors	KNN	K-nearest neighbor: no. neighbors K=1:2:26	70.54
	Discriminant analysis	LDA	Linear discriminant analysis	68.75
	Neural networks	MLP	Multilayer perceptron: no. h of hidden neurons: $h=h_0:\Delta h:h_1$, h0=max(1,$\lfloor h_1/H \rfloor$), H=10, Δh=max(1,$\lfloor (h_1-h_0)/H \rfloor$); h_1=max(1,$\lfloor N/(I+C) \rfloor$), N=no. train patterns, I=no. inputs, C=no. classes	66.96
	Regularized linear and logistic regression	Ridge	Ridge linear regression: regularization λ=1:5:200	67.86
		Logreg	Logistic regression	68.75
		SGD	Stochastic gradient descent: regularization =$\{10^{-5}, 5\cdot 10^{-5}, 10^{-4}, 5\cdot 10^{-4}$, 0.001, 0.005, 0.01, 0.05, 0.1, 0.5}	74.11
	Support vector machine	SVM	Support vector machine, RBF kernel: regularization $\lambda=\{2^i : i=-5:2:15\}$, spread $\gamma=\{2^i : i=-15:2:6\}$	70.54
	Bayesian	NB	Naive Bayes	52.68
**R** Several packages	Ensembles	Adaboost	Adaptive boosting (package *adabag*): no. trees T=50, 100; max. depth=1, 2	69.64
		Bagging	Bootstrap aggregating (*ipred*): no. trees T=10:2:56	67.86
		avNNet	Committee of multilayer perceptron neural networks (*caret*): no. hidden neurons h=1:9, weight decay=$\{0, 0.1, 0.01, 10^{-3}, 10^{-4}\}$	70.54
		RF	Random Forest (*randomForest*): no. random features F={2, 3, 4, 6, 7, 9, 10, 12, 13, 15}	70.54
	Neural networks	Nnet	Multilayer perceptron (*nnet*): : no. hidden neurons h=1:9, weight decay=$\{0, 0.1, 0.01, 10^{-3}, 10^{-4}\}$	64.29
		Neuralnet	Multilayer perceptron (*neuralnet*): no. hidden neurons h=1:6	63.39
	Decision trees	Ctree	Classification trees (*party*): max. depth=1:5, min. criterion={0.99, 0.745, 0.5, 0.255, 0.01}	68.75
		RPART	Recursive partitioning (*rpart*): complexity={0.05, 0.10, 0.15, 0.21, 0.26, 0.31, 0.42, 0.47}	65.18
	Discriminant Analysis	FDA	Flexible discriminant analysis (*mda*)	68.75
		LDA	Linear discriminant analysis (*MASS*)	68.75
	Nearest Neighbors	KNN	K-Nearest neighbors (*class*): no. of neighbors K=5:2:23	69.64
	Support vector machine	SVM	Support vector machine, RBF kernel (*kernlab*): regularization $\lambda=\{2^i : i=-4:0\}$, spread γ={0.02:0.01:0.07}	71.43
	Bayes	NB	Naive Bayes (*klaR*)	66.96

References

[i] Palm RB. Prediction as a candidate for learning deep hierarchical models of data. PhD. Thesis.

Technical University of Denmark, DTU Informatics. 2012. https://www2.imm.dtu.dk/pubdb/edoc/imm6284.pdf

[ii] Chang C, Lin C. LIBSVM : a library for support vector machines. *ACM Trans. Intel. Systs. Technol.* 2011;2(3):1–27. https://doi.org/10.1145/1961189.1961199

[iii] Fernández-Delgado M, Cernadas E, Barro S, Ribeiro J, Neves J. Direct Kernel Perceptron (DKP): Ultra-fast kernel ELM-based classification with non-iterative closed-form weight calculation, *Neural Netw.* 2014;50:60–71, https://doi.org/10.1016/j.neunet.2013.11.002.

[iv] Fung G, Mangasarian OL. Proximal Support Vector Machine Classifiers, *Proc. Knowledge Discovery and Data Mining.* August 26-29, 2001, San Francisco, CA. 77–86. https://doi.org/10.1145/502512.502527

[v] Wang Z, Shao YH, Wu TR. Proximal parametric-margin support vector classifier and its applications. *Neural Comput & Applic.* 2014;24: 755–764. https://doi.org/10.1007/s00521-012-1278-6

[vi] Littlestone N. Learning quickly when irrelevant attributes abound: a new linear-threshold algorithm, *Mach Learn.* 1988;2:285–318. https://doi.org/10.1109/SFCS.1987.37

[vii] Alateyat H, Fernández-Delgado M, Cernadas E, Barro S. Ultra fast classification and regression of high-dimensional problems projected on 2D. *Neural Process.* Lett. 2023;55:5377–5400. https://doi.org/10.1007/s11063-022-11090-3

[viii] Dorogush AV, Ershov V, Gulin A. CatBoost: gradient boosting with categorical features support. ArXiv pre-print 2018;1810.11363. https://arxiv.org/abs/1810.11363
